# Redistribution down under—dealing with ill-defined deaths in the Australian Burden of Disease Study

**DOI:** 10.1093/eurpub/ckac129.619

**Published:** 2022-10-25

**Authors:** M Dunford, W Ho, K Bishop, M Gourley, R Juckes

**Affiliations:** Population Health Group, Australian Institute of Health and Welfare, Canberra, Australia

## Abstract

Burden of disease analyses measure the healthy years of life lost due to living with and dying prematurely from disease and injury. It is now the global standard for comparable policy-relevant evidence on the impact of disease, injuries, and risks on a population. The Australian Burden of Disease Study (ABDS), undertaken by the Australian Institute of Health and Welfare, uses Australian-specific data and methods (based on the Global Burden of Disease Study, adjusted to suit the Australian context) to quantify disease burden for Australia. Despite the high quality of deaths registration data in Australia, in burden of disease analyses not all coded causes of death are considered appropriate or valid to estimate years of life lost (YLL). Therefore, these ‘ill-defined deaths’ are redistributed to one or more diseases on the ABDS disease list according to a more probable underlying cause of death. In the latest ABDS, almost 1 in 10 deaths in 2018 was an ill-defined death. Most of these were redistributed to other diseases using one of three methods developed for the Study:

1) direct evidence on more plausible causes of death from data linkage studies or other sources

2) redistribution algorithms based on the distribution of underlying causes of death where the ill-defined cause was recorded as an associated cause of death

3) reassignment of deaths across a specified range of target diseases according to patterns of causes of death observed in the mortality data for the ABDS disease list.

Expert advice was also received on the redistribution of deaths from septicaemia and deaths coded to ICD-10 code X59 Exposure to unspecified factor. Overall, 8.5% of the years of life lost for Australia in 2018 came from ill-defined deaths. These deaths generally followed age-specific all-cause mortality patterns. It is important to consider methods and target diseases when redistributing ill-defined deaths to appropriately quantify their contribution to disease-specific burden.

